# The effect of perioperative probiotics treatment for colorectal cancer: short-term outcomes of a randomized controlled trial

**DOI:** 10.18632/oncotarget.7045

**Published:** 2016-01-27

**Authors:** Yongzhi Yang, Yang Xia, Hongqi Chen, Leiming Hong, Junlan Feng, Jun Yang, Zhe Yang, Chenzhang Shi, Wen Wu, Renyuan Gao, Qing Wei, Huanlong Qin, Yanlei Ma

**Affiliations:** ^1^ Department of GI Surgery, Shanghai Tenth People's Hospital Affiliated to Tongji University, Shanghai, China; ^2^ Department of Surgery, Shanghai Jiao Tong University Affiliated Sixth People's Hospital, Shanghai, China; ^3^ Department of Pathology, Shanghai Tenth People's Hospital Affiliated to Tongji University, Shanghai, China

**Keywords:** colorectal cancer, perioperative probiotics treatment, post-operative outcome, randomised clinical trial

## Abstract

This study was designed to mainly evaluate the anti-infective effects of perioperative probiotic treatment in patients receiving confined colorectal cancer (CRC) respective surgery. From November 2011 to September 2012, a total of 60 patients diagnosed with CRC were randomly assigned to receive probiotic (*n* = 30) or placebo (*n* = 30) treatment. The operative and post-operative clinical results including intestinal cleanliness, days to first - flatus, defecation, fluid diet, solid diet, duration of pyrexia, average heart rate, length of intraperitoneal drainage, length of antibiotic therapy, blood index changes, rate of infectious and non-infectious complications, postoperative hospital stay, and mortality were investigated. The patient demographics were not significantly different (*p* > 0.05) between the probiotic treated and the placebo groups. The days to first flatus (3.63 *versus* 3.27, *p* = 0.0274) and the days to first defecation (4.53 *versus* 3.87, *p* = 0.0268) were significantly improved in the probiotic treated patients. The incidence of diarrhea was significantly lower (*p* = 0.0352) in probiotics group (26.67%, 8/30) compared to the placebo group (53.33%, 16/30). There were no statistical differences (*p* > 0.05) in other infectious and non-infectious complication rates including wound infection, pneumonia, urinary tract infection, anastomotic leakage, and abdominal distension. In conclusion, for those patients undergoing confined CRC resection, perioperative probiotic administration significantly influenced the recovery of bowel function, and such improvement may be of important clinical significance in reducing the short-term infectious complications such as bacteremia.

## INTRODUCTION

The amount of gut microbes, which may be nearly 10 times as many as host cells, fluctuates frequently and severely under the circumstances of many gut diseases, such as obesity, irritable bowel syndrome (IBS), inflammatory bowel disease (IBD), and even colorectal cancer (CRC) [1, 2]. Dysbiosis of gut bacteria generally occurs in cancer tissues which are directly exposed to microbes, such as colon and rectum [3, 4]. Recent studies confirmed the tight relationship between the microbiota imbalance and cancer progression [5–7]. For example, many opportunistic bacteria species, such as Helicobacter hepaticus, Streptococcus bovis, enterotoxigenic Eschericha coli (ETEC), enterotoxigenic Bacteroides fragilis (ETBF), and Fusobacterium nucleatum, are all confirmed to not only take responsibility for CRC carcinogenesis, but also affect clinical prognosis [1, 8].

Postoperative infection is a poor indicator for surgical treatment of cancer [9]. It occurs by a numbers of internal and external causes. Intestinal dysbiosis-induced bacterial translocation is the major driver of postoperative infection [9, 10]. Inappropriate use of antibiotics, chemotherapy, or even mechanical bowel preparation for patients undergoing confined colorectal cancer (CRC) resection operation, could also lead to microecological imbalance, subsequently exacerbating the risk for various infections [11]. Hence, probiotics biotherapy emerge as required.

Probiotics are beneficial bacteria that help sustain the homeostasis of gut microenvironment [12]. The ability of both the anti-infection and anti-carcinogen effect of probiotics are mainly based on the following: (a) mutagen binding, competitive inhibition and degradation; (b) host's innate and adaptive immunity enhancement; (c) beneficial gut microbes stimulation and metabolic activity improvement [13]. Therefore, oral administration of probiotics may be beneficial to the patients who are candidates for colorectal surgery [13, 14], however, the postoperative clinical benefit in maintaining balance of gut microbiota remains largely unexplored and unknown [15–19]. Our study was designed to primarily evaluate the anti-infective effects of perioperative probiotics treatment in patients receiving confined CRC resection operation.

## MATERIALS AND METHODS

### Ethics approval

Our study protocol was approved by the ethics committee of the Shanghai sixth People's Hospital affiliated to Shanghai Jiao Tong University. All participants were aware of the aim of this study and signed written informed consent prior to enrollment for random assignment. This trail was registered in www.chictr.org (Registrations number: ChiCTR-TRC-13003332) before participants recruitment started.

### Inclusion and exclusion criteria

Table 1 summarizes various patient inclusion and exclusion criteria for our study.

**Table 1:** Inclusion and exclusion criteria for patient enrollment in this clinical trial

Inclusion criteria	Exclusion criteria
Age 25 to 80;	Age more than 80 years old or younger than 25;
Received confined colorectal cancer resection operation;	Co-occurrence of other gastroenterological diseases (e.g. Inflammatory bowel disease);
Tolerated curative surgery;	Co-existence of other malignant neoplasms;
Diagnosed with sporadic colorectal cancer by biopsy examination and family history data collection;	Severe cardiovascular and cerebrovascular diseases that could not tolerate radical surgery;
No evidence of cancer metastasis;	Distant metastasis;
Joined the trial voluntarily and provided an informed consent.	Recent use of probiotics, prebiotics, or synbiotics;
N/A	Recent infection or recent antibiotic use;
N/A	Received emergency surgery or laparoscopic surgery;
N/A	Received neoadjuvant chemotherapy, radiotherapy, or biotherapy;
N/A	Evidence of Immunodeficiency;
N/A	Pregnancy.

N/A, not applicable.

### Participants

**Figure 1:**
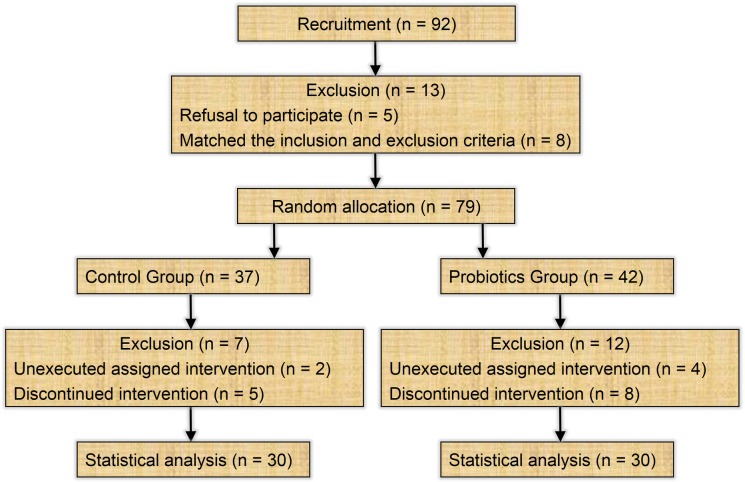
Flow diagram of the enrollment and randomization process

From November 2011 to September 2012, a total of 92 patients diagnosed with sporadic CRC at Shanghai Jiao Tong University Affiliated Sixth People's Hospital were recruited. After criteria eligibility determination, 13 patients were excluded by reason of either refusal to participate (*n* = 5) or matching the inclusion and exclusion criteria (*n* = 8). Of the 79 participants who were randomly assigned to control (*n* = 37) or probiotics group (*n* = 42), 7 and 12 patients, respectively, couldn't complete the study due to either unexecuted assigned intervention or discontinued intervention. At the end of the project, there were 60 eligible patients for statistical analysis, with 30 subjects in each group. Flow diagram of the enrollment and randomization process is illustrated in Figure 1.

### Study description

Randomization was performed in 1:1 proportion, according to a list of randomization numbers. Eligible patients received either probiotics or placebo treatment (2g, po, tid) for 12 consecutive days. Specifically, subjects who were assigned to the probiotics group received combined probiotics (Live combined Bifidobacterium, Lactobacillus and Enterococcus Power, Bifico, Sine Pharmaceuticals, Shanghai, China) containing *Bifidobacterium longum* (≥ 1.0*10^7^ cfu/g), *Lactobacillus acidophilus* (≥ 1.0*10^7^ cfu/g), and *Enterococcus faecalis* (≥ 1.0*10^7^ cfu/g) 5 days before and 7 days after CRC resection operation. This combined probiotics contains three acknowledged medicinal bacterial strain which function as maintaining healthy intestinal flora. When the oral administration was not feasible during the first day after surgery, the probiotics were administered via gastric gavage. Subjects who were assigned to the placebo group received placebo powder containing maltodextrin and sucrose, without any viable probiotics.

Both the probiotics and placebo drugs were packed in the same packaging and were appropriately stored at a controlled condition of 2°C to 8°C. All researchers and subjects were blinded to randomization and treatments during the entire interventional period. Conventional rehydration therapy without additional nutritional supplementations was given to all subjects during the study. For preoperative bowel preparation, all patients received a low-residue diet one day before operation and given 3 litres of polyethylene glycol electrolyte solution the night before surgery. For antibiotic prophylaxis, all patients received one dose of Cefoxitin 30 minutes prior to surgery. After operation, the antibiotic prophylaxis continued when the intraperitoneal drainage had not been removed or whenever the patients developed a fever of over 38.5°C. All subjects received open colorectal surgery by the same surgeon.

### Baseline variables and intraoperative indexes

The preoperative and intraoperative data of each subject were collected as follows: the gender, age, body mass index (BMI), tumor location, TNM stage, tumor differentiation, preoperative blood indices, length of operation, perioperative bleeding, and intraoperative gut cleanliness. The gut cleanliness scale was assessed according to the recommendation of the previous study [20]. The clinical demographics of eligible Participants are listed in Table 2.

**Table 2:** The clinico-pathological characteristics of enrolled participants

Characteristic	Placebo group (*n* = 30)	Probiotics group (*n* = 30)	*P* value
**Gender**			0.604
Female	18	15	
Male	12	15	
**Mean Age (years)**	62.17 ± 11.06	63.90 ± 12.25	0.567
**Body mass index (kg/m^2^)**	22.07 ± 1.70	22.13 ± 1.77	0.895
**Tumor location (n)**			
Left hemicolon	14	13	0.855
Right hemicolon	7	6	
Rectum	9	11	
**TNM stage (n)**			
0 - II	21	23	0.771
III	9	7	
**Tumor differentiation (n)**			
Well-moderate	27	25	0.707
Poor	3	5	
**Preoperative blood index**			
Leucocyte (*10^9^/L)	6.56 ± 2.54	6.14 ± 1.47	0.437
Hemoglobin (g/L)	116.20 ± 21.46	121.3 ± 22.08	0.371
Albumin (g/L)	38.5 ± 6.51	40.43 ± 5.41	0.216
Creatinine (umol/L)	67.1 ± 18.74	72.24 ± 22.85	0.345
Glucose (mmol/L)	5.43 ± 1.03	5.39 ± 0.67	0.858
Triglyceride (mmol/L)	1.56 ± 0.91	1.27 ± 0.50	0.353
Total cholesterol (mmol/L)	4.36 ± 0.98	3.95 ± 1.36	0.530
**Length of operation (min)**	128.34 ± 36.58	128.12 ± 28.31	0.979
**Perioperative bleeding (mL)**	116.60 ± 29.79	123.40 ± 30.50	0.388
**Intraoperative gut cleanliness (n)**			
Excellent	12	19	0.073
Good or fair	16	10	
Fair	2	1	

### Outcome variables

The pre-operative and post-operative clinical results, including intra-operative intestinal cleanliness, days to first - flatus, defecation, fluid diet, solid diet, duration of pyrexia, average heart rate, length of intraperitoneal drainage, length of antibiotic therapy, blood index changes, rate of non-infectious complications, infectious complications, postoperative hospital stay, and mortality, were compared. The primary endpoint was the influence of the diarrhea. The influence of the bowel function and the incidence of the other complications were set as secondary endpoints. Clinical definition of main complications is listed in Table 3.

**Table 3:** Clinical definition of complications

Complications	Clinical definition
**Pyrexia**	Oral temperature higher than 38.5°C.
**Infection**	
Bacteremia	7 day blood-cultures positive.
Wound infection	Surgical site suppuration and the bacterial cultures of purulent exudate positive.
Pneumonia	A typical pulmonary infiltrate can be seen on a chest X-ray and/or the swab culture is positive.
Urinary tract infection	There are obvious symptoms including frequent micturition, urgency to urinate, and urodynia, accompanied by bacteriuria (100,000 cfu/mL).
Anastomotic leakage	Presence of any of the following clinical signs: fecal discharge from the wound or drain, circumscribed abcess near the site of anastomosis, or fecal peritonitis confirmed by CT.
**Bowel dysfunction**	
Diarrhea	There is a symptom of having loose or liquid feces more than 3 times a day.
Abdominal distension	There is a sense of abdominal pressure or fullness.
**Mortality**	The occurrence of death during hospitalization

**Abbreviation:** cfu, colony-forming unit; CT, computed tomography.

### Statistical analysis

Statistical analysis were conducted using IBM SPSS Statistics 20.0 software (version 20.0, IBM, lnc., Chicago, IL, USA) and GraphPad Prism 6 software (version 6.01, GraphPad software, lnc., San Diego, CA, USA). We used the mean ± standard deviation (SD) to express the quantitative data. Unpaired Student's t-test was used to compare the continuous variables. Categorical variables were analyzed using the Pearson's Chi-square test and Fisher's exact test, as appropriate. Ordinal variables were analyzed using Mann-Whitney U test. A power of 80% and an alpha error of 5% were set to approximately calculated the sample size. All tests were double tail and the p values below 0.05 were considered statistically significant.

## RESULTS

A total of 30 participants were included in each group. The general demographics for the two groups of participants are presented in Table 2. There were no significant differences with regard to gender (*p* = 0.604), age (*p* = 0.567), BMI (*p* = 0.895), tumor location (*p* = 0.855), TNM stage (*p* = 0.771), and tumor differentiation (*p* = 0.707) between the probiotics and the placebo groups. Moreover, no significant differences were found between the two groups in term of the blood indices 5 days before surgery, including leucocyte count (*p* = 0.437), and levels of hemoglobin (*p* = 0.371), albumin (*p* = 0.216), creatinine (*p* = 0.345), glucose (*p* = 0.858), triglyceride (*p* = 0.353), and total cholesterol (*p* = 0.530), suggesting that the baseline of the two groups was quite homogeneous.

With regard to the intraoperative data, the length of operation (*p* = 0.979) and perioperative bleeding (*p* = 0.388) were similar between the two groups. It's worth mentioning that the intraoperative intestinal cleanliness had slight difference, although these results did not reach statistical significance (*p* = 0.073). This finding implied that perioperative probiotics treatment could likely be of tremendous clinical benefit as a supplement during bowel preparation in patients prepared for confined CRC surgery.

The Table 4 summarizes the information on postoperative short-term outcomes between the probiotics group and the placebo group. No significant differences (*p* > 0.05) were found between the placebo *vs*. probiotics groups in terms of days to first fluid diet (3.93 ± 0.78 *vs*. 3.73 ± 0.83 days; *p* = 0.341), days to first solid diet (5.00 ± 0.83 *vs*. 4.87 ± 0.86 days; *p* = 0.544), duration of pyrexia (4.80 ± 2.34 *vs*. 4.77 ± 1.79 days; *p* = 0.951), average heart rate in a week after surgery (78.98 ± 3.78 *vs*. 80.63 ± 4.13 bpm; *p* = 0.111), length of intraperitoneal drainage (6.67 ± 1.09 *vs*. 6.50 ± 0.97 days; *p* = 0.535), length of antibiotic therapy (7.33 ± 3.86 *vs*. 6.60 ± 2.81 days; *p* = 0.404), and postoperative hospital stay (15.00 ± 4.31 *vs*. 15.86 ± 4.92 days; *p* = 0.487). However, the days to first flatus (3.63 ± 0.67 days in the placebo group versus 3.27 ± 0.58 days in the probiotics group, *p* = 0.0274) and the days to first defecation (4.53 ± 1.11 days in the placebo group versus 3.87 ± 1.17 days in the probiotics group, *p* = 0.0268) were significantly improved in the probiotics group. These findings imply that the probiotics treatment resulted in a faster recovery of bowel function for patients with CRC operation.

**Table 4:** Postoperative short-term outcomes

Postoperative outcomes	Placebo group (*n* = 30)	Probiotics group (*n* = 30)	*P* value
**Days to first flatus (d)**	3.63 ± 0.67	3.27 ± 0.58	0.0274
**Days to first defecation (d)**	4.53 ± 1.11	3.87 ± 1.17	0.0268
**Days to first fluid diet (d)**	3.93 ± 0.78	3.73 ± 0.83	0.341
**Days to first solid diet (d)**	5.00 ± 0.83	4.87 ± 0.86	0.544
**Duration of pyrexia (> 38.5°C, d)**	4.80 ± 2.34	4.77 ± 1.79	0.951
**Average heart rate (in a week after surgery, bpm)**	78.98 ± 3.78	80.63 ± 4.13	0.111
**Length of intraperitoneal drainage (d)**	6.67 ± 1.09	6.50 ± 0.97	0.535
**Length of antibiotic therapy (d)**	7.33 ± 3.86	6.60 ± 2.81	0.404
**Postoperative hospital stay (d)**	15.00 ± 4.31	15.86 ± 4.92	0.487
**Blood index change**			
Leucocyte (*10^9^/L)	2.11 ± 2.26	1.64 ± 1.78	0.374
Hemoglobin (g/L)	14.73 ± 11.44	14.50 ± 10.58	0.935
Albumin (g/L)	7.93 ± 5.37	6.69 ± 4.40	0.336
Creatinine (umol/L)	14.13 ± 12.34	16.07 ± 11.36	0.534
Glucose (mmol/L)	1.39 ± 1.69	1.04 ± 1.36	0.541
Triglyceride (mmol/L)	0.35 ± 0.33	0.13 ± 0.04	0.136
Total cholesterol (mmol/L)	0.69 ± 0.63	0.79 ± 0.46	0.773
**Incidence of Infectious complications (%)**			
Bacteremia (%)	30.00 (9/30)	10% (3/30)	0.0528
Wound infection (%)	3.33 (1/30)	3.33 (1/30)	1.000
Pneumonia (%)	16.67 (5/30)	10.00 (3/30)	0.704
Urinary tract infection (n)	6.67 (2/30)	6.67 (2/30)	0.605
**Incidence of non-infectious complications (%)**			
Anastomotic leakage (%)	96.67 (1/30)	93.33 (2/30)	1.000
Diarrhea (%)	53.33 (16/30)	26.67 (8/30)	0.0352
Abdominal distension (%)	43.33 (13/30)	30.00 (9/30)	0.284
**Mortality (n)**	N/A	N/A	N/A

N/A, not applicable.

Blood indices related to blood routine and hepatorenal function was collected 5 days before and 7 days after surgery. All blood index changes were negative in the placebo group compared with the probiotics group, such as white blood cell (2.11 ± 2.26 *10^9^/L *vs*. 1.64 ± 1.78 *10^9^/L, *p* = 0.374), hemoglobin (14.73 ± 11.44 g/L *vs*. 14.50 ± 10.58 g/L, *p* = 0.935), albumin (7.93 ± 5.37 g/L *vs*. 6.69 ± 4.40 g/L, *p* = 0.336), creatinine (14.13 ± 12.34 umol/L *vs*. 16.07 ± 11.36 umol/L, *p* = 0.534), glucose (1.39 ± 1.69 mmol/L *vs*. 1.04 ± 1.36 mmol/L, *p* = 0.541), triglyceride (0.35 ± 0.33 mmol/L *vs*. 0.13 ± 0.04 mmol/L, *p* = 0.136), and total cholesterol (0.69 ± 0.63 mmol/L *vs*. 0.79 ± 0.46 mmol/L, *p* = 0.773).

We than compared the non-infectious and the infectious complications in the two groups. The incidence of diarrhea was significantly lower (*p* = 0.0352) in probiotics group (26.67%, 8/30) compared to the placebo group (53.33%, 16/30), whereas other non-infectious complications including anastomotic leakage (1 incident in the placebo group *vs*. 1 incidence in the probiotics group, *p* = 1.000), and abdominal distension (13 incidents in the placebo group *vs*. 9 incidents in the probiotics group, *p* = 0.284) were essentially quite comparable.

As for the infectious complications, although the incidence of bacteremia was slightly lower in probiotics group (30%, 9/30) than in placebo group (10%, 3/30), the difference didn't reach statistical significance (*p* = 0.0528). There were no statistical differences (*p* > 0.05) in other infectious complications rate. For example, the wound infection rate (1 incidence in each group respectively, *p* = 1.000) and the urinary tract infection rate (2 incidences in each group respectively, *p* = 0.605) were identical in the two groups. 5 Pneumonia complications occurred in the placebo group, whereas 3 incidents occurred in the probiotics group (*p* = 0.704). No side-effect of drug or mortality occurred in either group.

## DISCUSSION

The successfully use of probiotics have been reported to help administrate several functional gut disorders [14]. Thus, probiotics treatment for cancer-related gut complications would be of great interest. It is widely accepted that both surgical and nonsurgical treatments can frequently lead to gastrointestinal symptoms for patients with cancer [21]. Postoperative gastrointestinal symptoms such as diarrhea increase the risk of malnutrition, infectious complications, and longer hospitalization in patients with CRC surgery [20]. In our current study, we observed a significantly lower incidence of diarrhea (*p* = 0.0352) in probiotics group than in placebo group. In addition, in patients with the treatment of probiotics, the days to first flatus (*p* = 0.0274) or defecation (*p* = 0.0268) was earlier, implying faster recovery of bowel function. Such improvements may attribute to the effects of combined probiotics on the host physiology, including metabolism, intestinal function, bone homeostasis, and even emotion and behavior [1]. One of our recent study have already confirmed the effect of this combined probiotics on anti-inflammatory, regulation of immunity, and maintenance of gut barrier integrity in a interleukin-10 deficient mice model and Caco-2 cell line [22]. Our present study also confirmed the findings reported by a previous systematic reviews, which supported the potential benefit of probiotics in reducing diarrhea and sepsis rate in patients with cancer [15, 17, 23]. The relatively high incidence of diarrhea in our subjects may attribute to the open surgical proceure and high proportion of left semi-colorectal carcinoma.

Better bowel preparation may improve the disease outcome [24]. We found that after perioperative probiotics treatment, the intraoperative intestinal cleanliness had slight improvement. Whereas, one review study reported by peitsidou et al [19] did not advocate the combined use of beneficial microecologics and mechanical bowel preparation in patients with CRC operation. Thus, further studies, including larger patient cohort and longer probiotics use, are required to further confirm our results.

Several studies have assessed and supported the positive benefit for maintaining the intestinal microbiota balance by perioperative probiotics treatment in patients undergoing biliary Cancer Surgery, pancreatic duodenectomy, liver transplantation, and coloproctectomy [25–32]. Our previous study also supported the gut barrier protective function of perioperative probiotics treatment [33]. In our current study, the incidence of bacteremia was slightly lower in probiotics group than in placebo group (*p* = 0.0528). Our study suggests that, with short-term follow-up, perioperative probiotics administration significantly influenced the recovery of bowel function and such improvement may be of tremendous clinical value in reducing the infectious complications such as bacteremia or even gut-origin sepsis.

One limitation of this study was the relative short period of the use of probiotics. The other limitation was the absence of continuous administration of probiotics after hospital discharge in these subjects. It would be appropriate for follow-up to assess the benefits of long-term probiotics use for these subjects receiving chemotherapy.

## CONCLUSIONS

In conclusion, our study provides first-hand clinical evidence that perioperative probiotic administration may help those patients undergoing confined CRC resection surgery in obtaining short-term clinical benefit considering faster recovery of bowel function, lower incidences of diarrhea, and slightly lower rate of bacteremia.
